# Changes of placental three-dimensional power Doppler ultrasonography in third trimester among hypertensive disorders complicating pregnancy

**DOI:** 10.17305/bb.2023.9085

**Published:** 2023-10-01

**Authors:** Yingying Liu, Ximei Gao, Dandan Shi, Yong Wang, Xinying Qi, Na Wang

**Affiliations:** 1 Department of Obstetrics, Cangzhou Central Hospital, Cangzhou, China; 2 Medical Department, Cangzhou Central Hospital, Cangzhou, China

**Keywords:** 3D power Doppler ultrasonography, hypertensive disorders complicating pregnancy (HDCP), flow index (FI), vascularization index (VI), vascularization flow index (VFI)

## Abstract

Hypertensive disorders complicating pregnancy (HDCP) represent a systemic condition specific to pregnant women. Three-dimensional (3D) power Doppler ultrasonography is a technique that utilizes erythrocyte density, scattered intensity, or energy distribution in the bloodstream for imaging purposes. This study aimed to compare the changes in 3D power Doppler ultrasonography parameters in late pregnancy between patients with HDCP and those without HDCP, and to evaluate the predictive value of these parameters for pregnancy outcomes in patients with HDCP. The study included 160 pregnant women diagnosed with HDCP and 100 pregnant women without HDCP, who served as the control group. 3D power Doppler ultrasonography was performed, and the values of the vascularization index (VI), flow index (FI), and vascularization flow index (VFI) were measured. In the HDCP group, the VI, FI, and VFI were all lower than those observed in patients without HDCP. In HDCP patients with positive outcomes, these three parameters were higher than those recorded in patients with negative outcomes. The area under the predicted curve (AUC) for VI, FI, VFI, and the combination of these three parameters were 0.69, 0.63, 0.66, and 0.75, respectively. The parameters of 3D power Doppler ultrasonography can reflect the perfusion status of the placenta and predict the outcome of pregnancy in patients with HDCP. By monitoring these relevant hemodynamic parameters, valuable information can be provided for the clinical diagnosis, objective evaluation, and treatment of HDCP.

## Introduction

Hypertensive disorders complicating pregnancy (HDCP) is a systemic condition specific to mothers during pregnancy [1]. The prevalence of HDCP in China was 10.32% [2]. HDCP is clinically characterized by edema, hypertension, and proteinuria [3]. Severe HDCP is associated with headache and blurred vision, and may even lead to convulsions and coma [4]. HDCP, along with other pregnancy-related cardiovascular system disorders, poses a serious threat to maternal and fetal health [5]. Increased systemic vascular resistance in the mother impairs blood flow to many organs and can lead to serious complications [6]. If not properly treated, it can cause severe harm and even lead to the death of the pregnant woman and fetus. The etiology of HDCP has not been fully understood [7]. Treatment of hypertension in pregnancy is limited, so prevention and prediction of hypertension in pregnancy is particularly important [8].

Three-dimensional (3D) power Doppler ultrasonography is a technique that uses erythrocyte density, scattered intensity, or energy distribution in the bloodstream for imaging [9]. The technique is relatively simple and safe because it is noninvasive and does not require radiation exposure [10]. 3D power Doppler ultrasonography provides a qualitative assessment of vessel type and distribution characteristics [11]. It is possible to use the sensitivity of this method for low velocity blood flow and analyze blood flow and resistance conditions in the placenta [12]. It has been demonstrated that 3D power Doppler ultrasonography can quantify placental perfusion in the anterior wall and provide a quantitative assessment of placental perfusion for the diagnosis of HDCP [13]. However, specific changes in 3D power Doppler ultrasonography parameters in patients with HDCP in late pregnancy and their association with pregnancy outcome in HDCP patients have not yet been reported. In this study, we compared the changes in 3D power Doppler ultrasonography parameters during late pregnancy in HDCP patients and non-HDCP pregnant women and evaluated the predictive value of these parameters for pregnancy outcome in HDCP patients.

## Materials and methods

### Participants

In this study, 160 pregnant women with HDCP were included, whereas 100 pregnant women without HDCP served as the control group. Both groups were followed until the end of their pregnancies. All participants registered prenatally in our obstetrics department, delivered at our hospital, and signed an informed consent form.

The diagnostic criteria for HDCP were in accordance with the 2013 American College of Obstetricians and Gynecologists (ACOG) guidelines for hypertension in pregnancy, including screening and exclusion criteria.

The diagnostic criteria for preeclampsia (PE) were: after 20 weeks of gestation, if normal previous blood pressure present, a systolic blood pressure (SBP) of ≥ 140 mmHg measured at four-h intervals or a diastolic blood pressure (DBP) of ≥ 90 mmHg; 24-h urine protein ≥ 300 mg (or estimated by collecting urine for a limited time) or urine protein/creatinine ≥ 0.3; without present proteinuria but present liver dysfunction, pulmonary edema, renal dysfunction, thrombocytopenia, cerebral or visual disturbances.

The diagnostic criteria for severe PE were, based on PE, accompanied by any of the following symptoms: DBP ≥ 110 mmHg or SBP ≥ 160 mmHg (interval four hours, without prior use of antihypertensive medication); platelets < 100.000/µL; abnormal liver function (elevated transaminases, persistent severe pain in the right upper quadrant or below the diaphragm); progressive renal dysfunction; pulmonary edema; new brain damage and visual disturbances.

The inclusion criteria for HDCP patients were: HDCP pregnant women diagnosed in the third trimester according to the above diagnostic criteria (HDCP patients were included in the third trimester, but the first diagnosis was not limited to the third trimester); age over 18 years; singleton pregnancy.

Exclusion criteria for HDCP patients were: inadequate localization of the placenta, including the anterior and posterior placentas; hypertension before pregnancy; multiple pregnancy; diabetes, cerebrovascular, and severe hepatic and renal insufficiency; concomitant acute and chronic injuries, infections, and rheumatism, rheumatoid, and other immune system-related diseases.

Inclusion criteria for pregnant women without HDCP were: pregnant women in the third trimester; age over 18 years; singleton pregnancy; without HDCP.

The exclusion criteria for pregnant women without HDCP were: Pre-pregnancy hypertension; multiple pregnancy; diabetes, cerebrovascular and severe hepatic and renal insufficiency; concomitant acute and chronic injuries, infections and rheumatism, rheumatoid and other immune system-related diseases.

### 3D power Doppler ultrasonography

**Figure S1.**
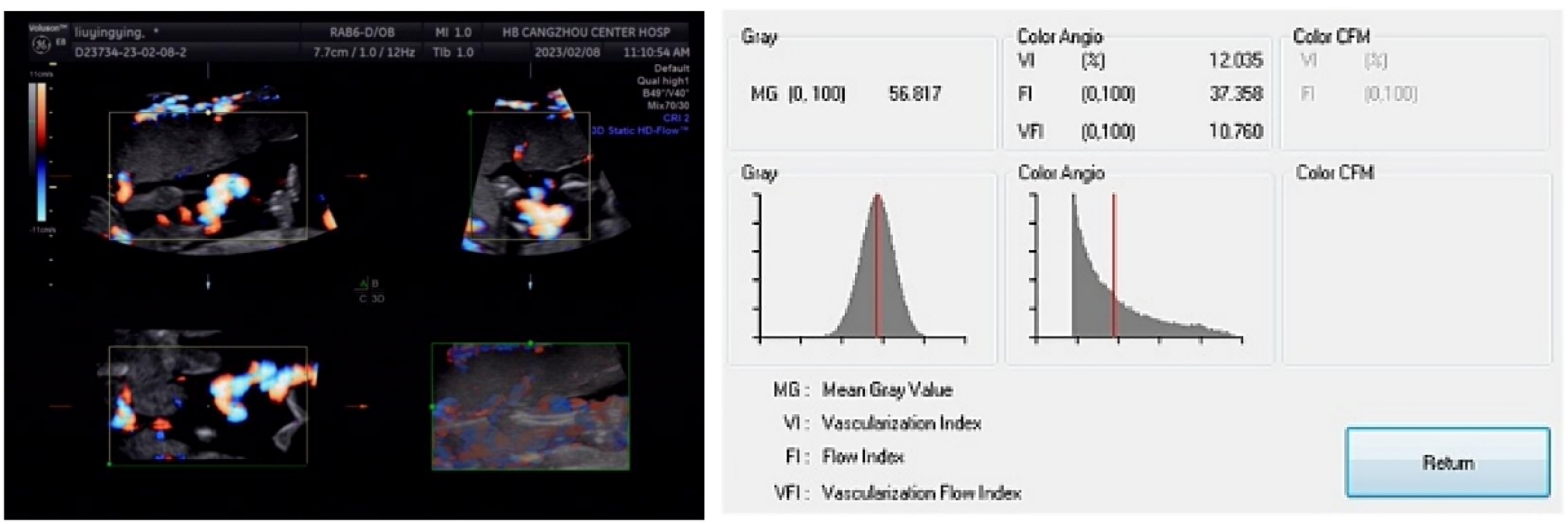
**Representative spherical contour of the placenta measured by three-dimensional power Doppler ultrasonography and the output of the vocal analysis.** CFM: Color flow mode.

A Voluson E8 (GE Medical Systems, Milwaukee, WI, USA) ultrasound system was used. In addition to routine ultrasound, the placental 3D power Doppler was performed in all women. The five spherical sampling sites in each placenta were as follows: one central, two peripheral at opposite sides, and one between the central site and each peripheral site. The peripheral sampling spheres were always positioned to maintain a minimum distance of approximately 4 mm between the insonated region and the chorionic and basal plates. Maximum sensitivity was ensured with the following settings: pulse repetition frequency 0.6 kHz; wall motion filter low 1; frequency medium; dynamic 3; balance 4150; smooth 4/5; ensemble 11; line density 8; power Doppler map 4; artifact suppression off; power Doppler line filter off; quality high. The placenta was examined with a constant volume and the same 25-degree sector angle. The spheres were kept away from the spiral arteries and placed near the basal and/or chorionic plate. The 3D glass body render mode was used, in which the color and gray-scale information was processed and combined to form a 3D image. Vascularization indices were determined using the virtual organ computer-aided analysis (VOCAL) program. Manual mode was set, and the five spherical regions of interest in each placenta were manually circled by rotating 30 degrees six times, respectively. The placenta was examined with a constant volume and the same 25-degree sector angle. The spheres were kept away from the spiral arteries and placed near the basal and/or chorionic plate. The volume of spheres did not contain vessels of the basal and chorionic plates and was kept constant. A histogram automatically displayed the vascularization indices. The 3D volume was formed using basic units called voxels. The voxel contains all the information about the grayscale and color, according to an intensity scale ranging from zero to 100. The vascularization indices were as follows: the vascularization index (VI) refers to the ratio of color voxel to total voxel and indicates the number of vessels that could be detected within the placental volume; the flow index (FI) refers to the weighted color voxel divided by the ratio of total color voxels and provides an amplitude value for the color signal that indirectly estimates blood flow in the placenta during a 3D sweep; the vascularization flow index (VFI) refers to the weighted ratio of color voxels to total voxels, indicating a combination of vascularity and blood flow. The representative spherical contour of the placenta measured by 3D power Doppler ultrasonography and the output of vocal analysis is shown in Figure S1.

### Ethical statement

The study was approved by the ethics committee of Cangzhou Central Hospital (CZ.9240.v67).

**Table 1:** Demographic and clinical characteristics of pregnant women with HDCP and without HDCP (control)

**Characteristics**	**Control (*n* ═ 100)**	**HDCP (*n* ═ 160)**	***p* value**
Maternal age (years)	29.4 ± 3.8	30.1 ± 4.2	0.195
Gestational weeks at admission	34.8 ± 3.9	35.2 ± 3.6	0.251
BMI before pregnancy (kg/m^2^)	22.9 ± 2.7	23.5 ± 3.4	0.137
Proteinuria (mg/24h)	65.8 ± 42.1	296.8 ± 114.6	<0.001
SBP (mmHg)	113.7 ± 10.9	146.4 ± 13.1	<0.001
DBP (mmHg)	78.6 ± 8.2	103.1 ± 9.8	<0.001
*Parity (n, %)*			
Nulliparous	68 (68)	97 (60.6)	0.237
Multiparous	32 (32)	63 (39.4)	
*Education status (n, %)*			
Junior high school and below	12 (12)	24 (15)	0.709
Senior high school or polytechnic school	41 (41)	59 (36.9)	
College and above	47 (47)	77 (48.1)	
*Clinical classification (n, %)*			
GH	–	72 (45)	**–**
mPE		51 (31.9)	
sPE		37 (23.1)	

The data presented are mean ± standard deviation or *n* (percentage). The comparisons of data between the two groups were done by Mann–Whitney test or Fisher’s exact test or chi-square test. HDCP: Hypertensive disorders complicating pregnancy; BMI: Body mass index; SBP: Systolic blood pressure; DBP: Diastolic blood pressure; GH: Gestational hypertension; mPE: Mild preeclampsia; sPE: Severe preeclampsia.

### Statistical analysis

**Figure S2.**
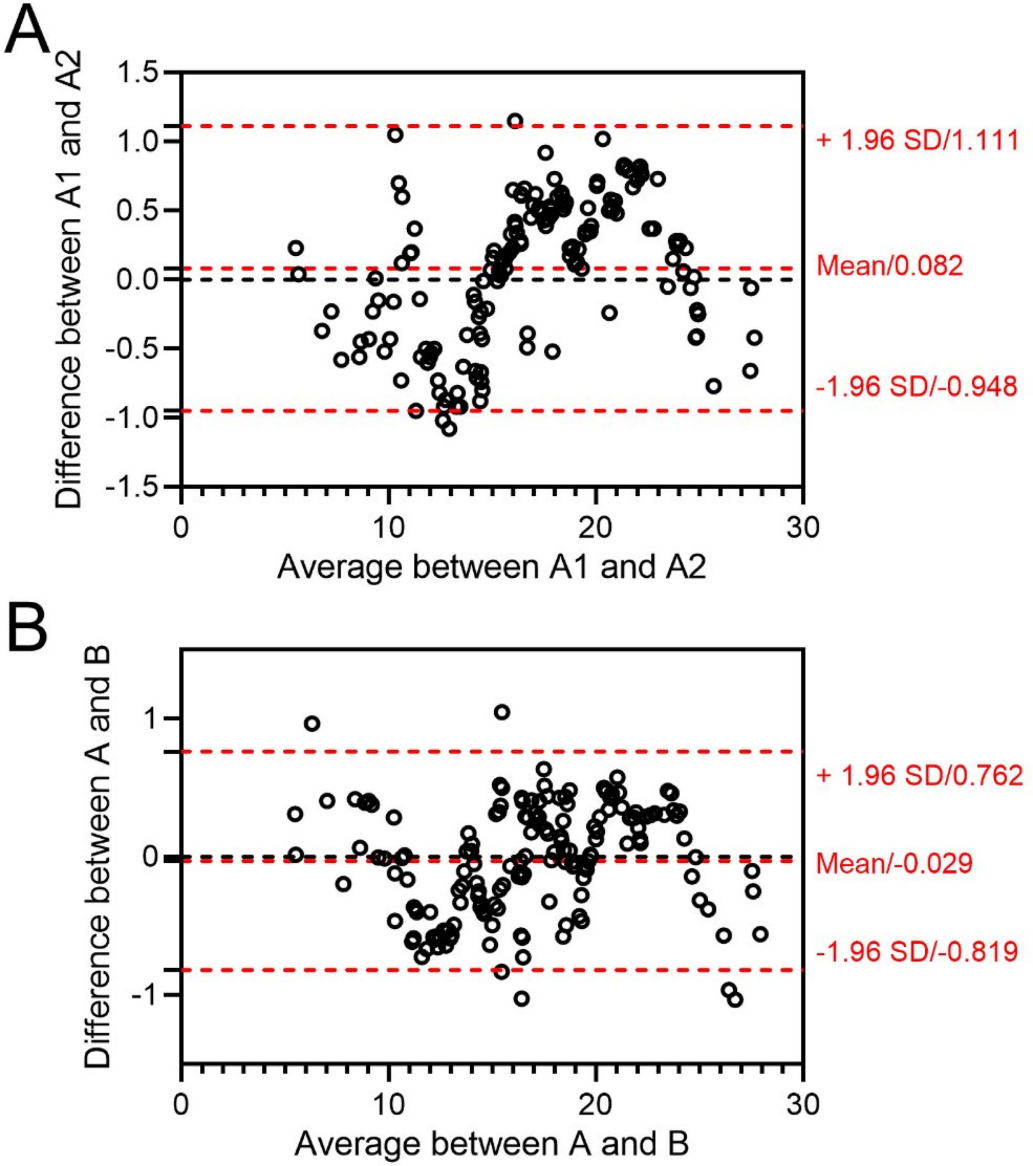
**Bland–Altman plots of present intra- and inter-observer reproducibility and reliability of vascularization index in hypertensive disorders complicating pregnancy** **group**. A and B: Two observers; 1, 2: Double analysis for each observer; SD: Standard deviation.

In this study, VI, FI and VFI values were obtained from each sample measurement, and the average value of the three indexes from three measurements was used for the final statistical analysis. 3D power Doppler ultrasonography images were collected and analyzed by experienced physicians who were blinded to the experimental grouping. To validate the present intra- and inter-observer reproducibility and reliability, we asked two additional observers to perform a reanalysis of the VI data for the HDCP group. The two observers were designated A and B and each repeated the analysis twice, designated 1 and 2. The Bland–Altman plot showed good measurement consistency between the two observers and good interobserver repeatability. Moreover, the correlation coefficients between A1 and A2 were 0.995 (0.993–0.996) and the correlation coefficients between A and B were 0.996 (0.995–0.997) (Figure S2). The data analysis was conducted with SPSS version 23 for Windows (SPSS Inc., Chicago, IL, USA). The data presented are mean ± SD or n (percentage). The comparisons of data were performed by Fisher's exact test, unpaired *t*-test with Welch's correction, or one-way ANOVA followed by Dunn's multiple comparisons test. The predictive values of the parameters were determined by ROC curves. Cut-off value was calculated based on the Youden index. Logit (Combination) ═ −0.155 * VI −0.043 * FI −0.156 * VFI. *p* value < 0.05 was considered significant.

## Results

The characteristics of the pregnant women are shown in Table 1, which reveals no significant difference between the control group and the HDCP group in maternal age, weeks of gestation at admission, pre-pregnancy body mass index (BMI), parity, and educational status. However, proteinuria, SBP, and DBP were significantly higher in the HDCP group. Of the 160 patients with HDCP, 72 had gestational hypertension (GH group), 51 had mild PE (mPE group), and 37 had severe PE (sPE group).

The pregnancy outcomes of the pregnant women are shown in Table 2. The cases of prematurity, puerperal infection, postpartum hemorrhage, nonreassuring fetal status, neonatal asphyxia, placental abruption, and fetal growth restriction were significantly higher in the HDCP group. However, there was no significant difference in premature rupture of membranes between the two groups.

**Table 2:** Comparisons of pregnant outcomes between pregnant women with HDCP and without HDCP (control)

	**Study group**		
**Characteristics**	**Control (*n* ═ 100) *n* (%)**	**HDCP (*n* ═ 160) *n* (%)**	***p* value**
Prematurity	9 (9)	35 (21.9)	0.007
Puerperal infection	3 (3)	18 (11.3)	0.019
Postpartum hemorrhage	4 (4)	21 (13.1)	0.017
Nonreassuring fetal status	2 (2)	19 (11.9)	0.004
Neonatal asphyxia	1 (1)	13 (8.1)	0.012
Premature rupture of membranes	3 (3)	9 (5.6)	0.381
Placental abruption	0 (0)	7 (4.4)	0.046
Fetal growth restriction	3 (3)	16 (10)	0.048
Total poor outcomes	13 (13)	72 (45)	<0.001

The comparison of data between the two groups was done by Fisher’s exact test. HDCP: Hypertensive disorders complicating pregnancy.

**Figure 1.**
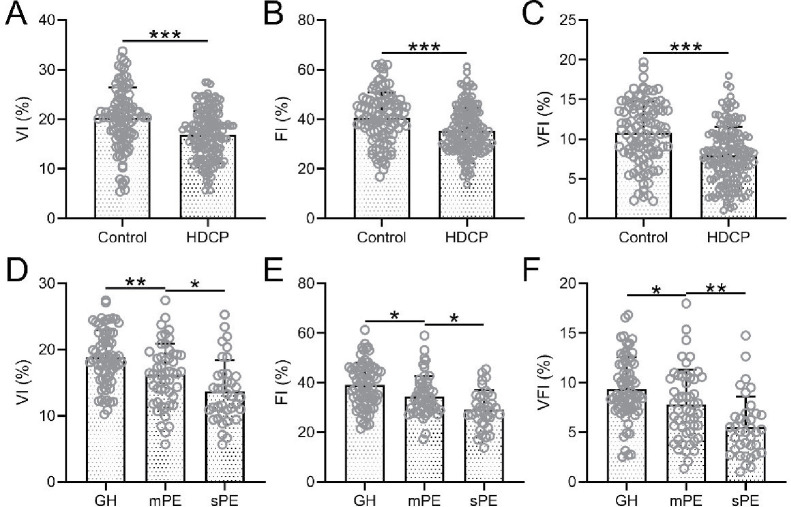
**Comparisons of placental three-dimensional power Doppler ultrasonography parameters in the third trimester, including VI (A), FI (B), and VFI (C) between normotensive (control, *n* ═ 100) and HDCP (*n* ═ 160).** ***p < 0.001, Unpaired *t*-test with Welch’s correction. *p < 0.05, **p < 0.01, One-way ANOVA followed by a Dunn’s multiple comparisons test. Comparisons of placental three-dimensional power Doppler ultrasonography parameters in the third trimester VI (A), FI (B), and VFI (C) among GH (*n* ═ 72), mPE (*n* ═ 51), and sPE (*n* ═ 37). VI: Vascularization index; FI: Flow index; VFI: Vascularization flow index; HDCP: Hypertensive disorders complicating pregnancy; GH: Gestational hypertension; mPE: Mild preeclampsia; sPE: Severe preeclampsia.

In patients in the HDCP group, VI, FI, and VFI were significantly lower than those without HDCP (Figure 1A–1C). These three parameters were significantly lower in the mPE group than in the GH group, and they were significantly lower in the sPE group than in the mPE group (Figure 1D–1F).

**Figure 2.**
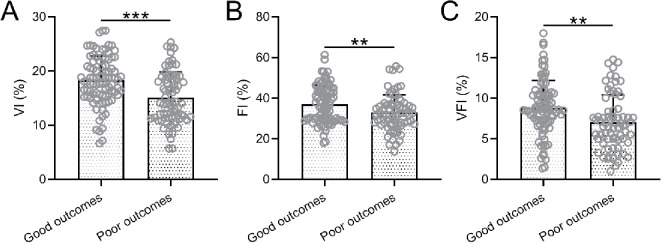
**Comparisons of placental three-dimensional power Doppler ultrasonography parameters in the third trimester, including VI (A), FI (B), and VFI (C) between good pregnant outcomes (*n* ═ 88) and poor pregnant outcomes (*n* ═ 72) among pregnant women with hypertensive disorders complicating pregnancy.** **p < 0.01, ***p < 0.001, Unpaired *t*-test with Welch’s correction. VI: Vascularization index; FI: Flow index; VFI: Vascularization flow index.

Of the 160 patients with HDCP, 72 had a poor outcome. In the HDCP patients with good outcomes, VI, FI, and VFI were all significantly higher than in those patients with poor outcomes (Figure 2A–2C).

**Table 3:** Predictive values in ROC analysis.

	**Cut-off value**	**AUC (95% CI)**	***p* value**	**Sensitivity (%)**	**Specificity (%)**	**Youden index**
VI	14.3	0.69 (0.60–0.77)	<0.001	54.17	84.09	0.38
FI	37.66	0.63 (0.54–0.71)	0.006	79.17	48.86	0.28
VFI	7.71	0.66 (0.57–0.75)	<0.001	72.22	67.05	0.39
Combination^*^	–	0.75 (0.68–0.83)	<0.001	77.78	70.45	0.48

^*^Logit (Combination) ═ −0.155 * VI-0.043 * FI-0.156 * VFI. ROC: Receiver operating characteristic; AUC: Area under the curve; CI: Confidence interval; VI: Vascularization index; FI: Flow index; VFI: Vascularization flow index.

**Figure 3.**
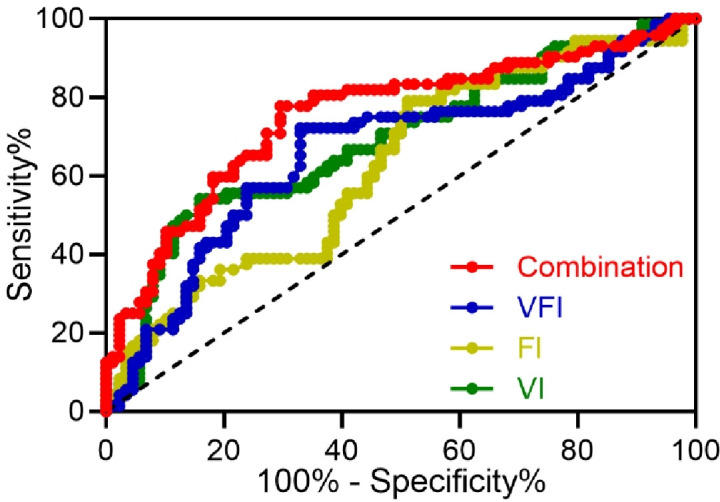
**ROC analysis of predictive values of placental three-dimensional power Doppler ultrasonography parameters in the third trimester for pregnant outcomes among pregnant women with hypertensive disorders complicating pregnancy.** VFI: Vascularization flow index; FI: Flow index; VI: Vascularization index; ROC: Receiver operating characteristic.

The area under the curve (AUC) of VI in predicting pregnancy outcome in pregnant women with HDCP was 0.69 (95% CI 0.60–0.77), with a sensitivity of 54.17% and a specificity of 84.09% (Figure 3 and Table 3). The AUC of FI for predicting pregnancy outcome in pregnant women with HDCP was 0.63 (95% CI 0.54–0.71), with a sensitivity of 79.17% and a specificity of 48.86% (Figure 3B and Table 3). The AUC of VFI for predicting pregnancy outcome in pregnant women with HDCP was 0.66 (95% CI 0.57–0.75), with a sensitivity of 72.22% and a specificity of 67.05% (Figure 3B and Table 3). The AUC of the combination of these three parameters for predicting pregnancy outcome in pregnant women with HDCP was 0.75 (95% CI 0.68–0.83), with a sensitivity of 77.78% and a specificity of 70.45% (Figure 3C and Table 3).

## Discussion

Hypertension in pregnancy is a unique obstetric complication that affects pregnant women. Its main features include widespread vasoconstriction and impaired uteroplacental perfusion, which can lead to serious maternal and fetal complications and affect the lives of mothers and children [14]. According to studies, the worldwide incidence of hypertension during pregnancy is approximately 10%, and 13% of maternal deaths attributable to direct obstetric factors are related to PE [1]. The main clinical manifestations of hypertension in pregnancy are elevated blood pressure with or without proteinuria and edema. If the disease progresses rapidly, convulsions and coma may occur [15]. Currently, the etiology and pathogenesis of hypertension in pregnancy are thought to be a combination of fetal, placental, and maternal factors [16]. Early determination and prediction of the severity of hypertension in pregnancy may help to reduce the complication rate and mortality of maternal and perinatal diseases and is therefore of great value.

Since the application of 3D power Doppler ultrasonography, its increasing color sensitivity, and combined use with computerized software have enabled quantitative analysis of placental blood perfusion [17, 18]. Placental vascular sonobiopsy using 3D power Doppler ultrasound is used to assess placental vascularization in normal and growth-restricted fetuses [19–21]. 3D power Doppler ultrasound also shows that the vascularity and flow intensity of the placenta in normal pregnancy are different from those in PE [22, 23].

The aim of this study was to compare the changes in 3D power Doppler ultrasonography parameters VI, FI, and VFI between HDCP patients and non-HDCP pregnant women in late pregnancy and to evaluate the predictive value of these parameters for pregnancy outcome in HDCP patients. These results may provide a valid clinical assessment index to reduce perinatal mortality.

Several studies have confirmed that the pathological basis of HDCP is systemic spasm of small arteries, which impairs microvascular circulation and increases capillary permeability [24]. Maternal hypertensive disease may lead to an increase in blood volume and pressure in the placental circulation, resulting in acute necrotizing arteritis and vascular embolism, reducing the effective exchange area and causing ischemia and hypoxia in the placental tissue. In severe cases, local tissue hemorrhage, necrosis, infarction, and circulatory disorders between the fetus and the placenta may also occur [25].

Placenta is a special and important organ between mother and fetus, and its function directly affects the health of the fetus. However, intra-placental blood flow velocity is low, and color Doppler ultrasonography cannot effectively monitor the intra-placental vascular tree because of the direction of blood flow [26]. In contrast, 3D power Doppler ultrasonography can visualize low-velocity blood flow and is independent of the direction of blood flow, which clearly reveals the tertiary structure of intra-placental villi vessels [27]. VI, FI, and VFI are new indicators of placental blood flow assessed by 3D power Doppler ultrasonography [11]. All three indices are significantly affected by volume flow, attenuation, vessel number, and erythrocyte density [28]. In normal pregnancies, all placental vascular indices estimated by 3D power Doppler ultrasonography had a constant distribution throughout pregnancy [29]. VI represents the number of vessels detected per unit volume and is used to indicate the sparseness of vessel distribution. FI is the number of blood cells passed during the 3D energy scan and VFI is the combination of VI and FI. Studies have shown that as the gestational week increases, blood vessels in the placenta enlarge and thicken, blood flow increases, and VI, FI, and VFI also increase [30]. It has been reported that placental vascular indices in 3D power Doppler measurements are influenced by the location of placental implantation [18]. The position of the placental attachment in the uterine cavity leads to a significant decrease in the individual indices as the distance between the transducer and the vessel increases [28]. Some studies have shown that only the anterior placentas should be included in studies to avoid the risk of Doppler signal attenuation [19, 31]. Therefore, patients with inadequate placental localization, including anterior and posterior placentas, were excluded in this study.

After comparing the 3D power Doppler ultrasonography parameters of the placenta VI, FI, and VFI in late pregnancy between control and HDCP patients, we found that all three parameters were significantly decreased in patients with HDCP. We then compared the differences in these three parameters between patients with different severity of HDCP and observed a decreasing trend in VI, FI, and VFI with increasing severity of HDCP.

Based on Table 2, we analyzed prematurity, puerperal infection, postpartum hemorrhage, nonreassuring fetal status, neonatal asphyxia, placental abruption, fetal growth restriction, and premature rupture of membranes in patients with HDCP and confirmed that 72 patients (45%) had adverse pregnancy outcomes. When comparing the 3D power Doppler ultrasonography parameters of late pregnancy VI, FI, and VFI between the poor-outcome and good-outcome groups, it was found that all three parameters were significantly reduced in the poor-outcome group.

In this study, we analyzed the predictive value of 3D power Doppler ultrasonography parameters for pregnancy outcome in HDCP patients using ROC and determined the cut-off value based on the maximum value of the Youden index. The results showed that the 3D power Doppler ultrasonography parameters VI, FI, and VFI of the placenta in late pregnancy were effective in predicting pregnancy outcome in patients with HDCP, and the combination of the three parameters significantly improved the Youden index.

However, this study had some limitations. Although the total population of the study was relatively large, the small sample size of patients with gestational hypertension, mild PE, and severe PE may have reduced the power of this study. In addition, this study did not collect long-term outcome data for the offspring. In addition, differences in placental flow were not compared with individual abnormal outcomes, and sensitivity, specificity, and AUC analysis did not show ideal accuracy, which needs to be further verified in future larger-scale studies. Because our study population included only women from a single tertiary center, our data may not be representative of the entire population.

## Conclusion

Placental hemodynamics are altered in patients with HDCP, which seriously affects maternal and fetal health. This study suggests that 3D power Doppler ultrasonography parameters can reflect the perfusion status of the placenta and predict pregnancy outcome in patients with HDCP. By monitoring the relevant hemodynamic parameters, valuable information can be obtained for clinical diagnosis, objective evaluation, and treatment of HDCP.

## Supplemental Data
